# Early Detection of Gastric Cancer: Linking Epidemiology, Pathophysiology, and Innovations in Digestive Endoscopy

**DOI:** 10.3390/diseases14040148

**Published:** 2026-04-18

**Authors:** Marta La Milia, Mario Capasso, Tommaso Pessarelli, Guido Manfredi, Arnaldo Amato

**Affiliations:** 1 Digestive Endoscopy and Gastroenterology Department, Ospedale A. Manzoni, 23900 Lecco, Italy; m.lamilia@asst-lecco.it (M.L.M.); a.amato@asst-lecco.it (A.A.); 2 Gastroenterology and Endoscopy Unit, ASST Ospedale Maggiore, 26013 Crema, Italyguido.manfredi@asst-crema.it (G.M.)

**Keywords:** gastric cancer, gastric dysplasia, gastric intestinal metaplasia, digestive endoscopy, quality in digestive endoscopy, epidemiology, chromoendoscopy, virtual chromoendoscopy, acetic acid

## Abstract

Background/Objectives: Despite substantial progress in understanding its pathophysiology and risk factors, gastric cancer remains a significant global health burden. Advances in endoscopic technology have improved the potential for early detection, yet variability in clinical practice persists. In this comprehensive narrative review, we summarize the most recent epidemiological trends in gastric pre-neoplastic and neoplastic lesions and critically appraise current evidence on optimizing endoscopic techniques and strategies for the detection of early gastric neoplasia, with an emphasis on emerging innovations. Methods: The relevant literature on epidemiology, risk factors, pathophysiology, and endoscopic management of GC was selectively reviewed based on the authors’ expertise and appraisal of contemporary evidence. Results: Marked global disparities persist in GC incidence, mortality, and stage at diagnosis. Interval GC—including missed lesions and so-called “true” interval cancers—remains a clinically relevant challenge and is frequently identified at advanced stages. These gaps are partly attributable to inconsistent quality in diagnostic esophagogastroduodenoscopy (EGD). High-quality EGD relies on adequate mucosal inspection time, systematic photodocumentation, optimal gastric preparation, and the use of standardized terminology, including mucosal visibility scores. Routine integration of chromoendoscopy and magnification techniques further enhances detection rates. Looking ahead, artificial intelligence holds promise as a transformative adjunct to standardize and augment real-time lesion recognition and quality assurance. Conclusions: High-quality endoscopic evaluation, coupled with tailored surveillance strategies, enables earlier detection of pre-neoplastic lesions and early gastric cancer, improving clinical outcomes. Future priorities include broadening access to high-quality endoscopy, harmonizing performance standards, and promoting continuous training alongside technological integration.

## 1. Introduction

Gastric cancer (GC) remains a major global health burden, ranking among the leading causes of cancer-related morbidity and mortality worldwide. Despite a gradual decline in incidence in several Western countries, GC is frequently diagnosed at an advanced stage, resulting in poor prognosis and limited therapeutic options [1]. Despite this decreasing trend, the total number of cases is expected to increase due to the increasing population aging and demographic expansion in countries with a high GC incidence [2]. Over the past decades, substantial progress has been made in understanding the epidemiology, pathophysiology, and etiological factors of GC, particularly the central role of Helicobacter pylori (HP) infection and chronic inflammation in gastric carcinogenesis [3]. Unlike many other solid malignancies, GC typically develops through a well-recognized multistep cascade, progressing from chronic gastritis to atrophic gastritis, intestinal metaplasia, dysplasia, and ultimately invasive carcinoma [4]. Pre-neoplastic and early neoplastic lesions are potentially detectable during esophagogastroduodenoscopy (EGD), offering a crucial opportunity for early diagnosis and curative treatment. Concurrently, digestive endoscopy has undergone remarkable evolution, driven by technological innovations, such as high-definition endoscopy, image-enhanced endoscopy, and magnification techniques, as well as by increasing endoscopist expertise. Growing evidence indicates that high-quality endoscopic examination, combined with appropriate surveillance strategies, enables the detection of a substantial proportion of pre-neoplastic lesions and early gastric cancer (EGC), significantly improving patient outcomes [5]. Nevertheless, the incidence of advanced GC and the rate of those diagnosed after a previously negative upper endoscopy remain unacceptably high [6]. These findings highlight persistent limitations in real-world endoscopic practice, including variability in examination quality, lesion recognition, and adherence to surveillance recommendations. In this narrative comprehensive review, we aim to summarize the most recent epidemiological data on gastric pre-neoplastic and neoplastic lesions and to critically summarize current evidence on optimizing endoscopic techniques and strategies to detect early gastric neoplasia.

## 2. Epidemiology of Gastric Neoplastic Lesions: The Submerged Diagnosis

### 2.1. Gastric Cancer

**Figure 1:**
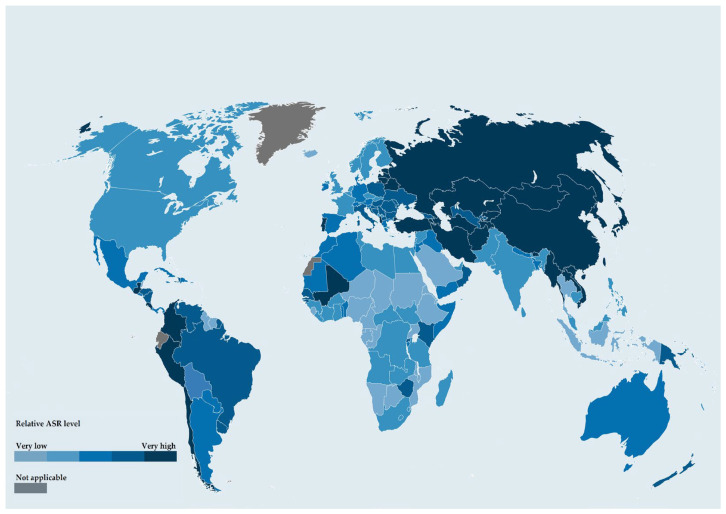
Global distribution of gastric cancer (GC) incidence in 2022 according to age-standardized rates (ASR) per 100,000 inhabitants. Countries are classified into categories according to ASR levels, ranging from very low (such as Middle Africa, ASR 3.3) to very high (such as Eastern Asia, with ASR reaching 23.0); grey areas indicate countries with no available data. Adapted from GLOBOCAN 2022 estimates [8].

GC remains a major global health challenge and represents a substantial contributor to cancer-related morbidity and mortality worldwide. In 2017, it accounted for more than one million new cases, nearly 865,000 deaths, and approximately 19 million disability-adjusted life-years (DALYs), underscoring its considerable population-level impact [7]. Recent worldwide data report an age-standardized incidence of 9.2 per 100,000 person-years, with considerable regional variability (Figure 1) [8]. Less than a century ago, GC was the most common malignancy globally; although incidence and mortality have markedly declined in many regions, emerging evidence suggests a potential reversal of this long-standing downward trend. Several studies from the United States indicate rising incidence among individuals younger than 50 years, a phenomenon that may eventually counterbalance reductions achieved in older age groups [9,10]. More than 90% of GCs are adenocarcinomas, subdivided into cardia and non-cardia types according to their anatomical location relative to the gastro-esophageal junction [11]. The historical decline in GC incidence has been driven primarily by reductions in non-cardia cancer, closely linked to decreasing HP prevalence [12]. Indeed, HP is a well-established carcinogen for non-cardia GC. In the past, HP infected most adults worldwide [13], while improvements in living conditions, antibiotic use and targeted eradication strategies during the second half of the twentieth century may have contributed to the marked decline in infection rates observed globally [14]. However, HP infection is far from becoming rare, with recent data showing a worldwide prevalence of 44.3%, ranging from 50.8% in developing countries to 34.7% in developed countries [15]. In terms of HP infection, the epidemiology of GC shows pronounced geographical heterogeneity, with incidence varying by as much as 5–10-fold between high-risk and low-risk regions [16]. Eastern Asia, parts of Eastern Europe, and several South American countries continue to experience the highest incidence rates. While these differences partly reflect variability in HP infection, environmental and lifestyle factors also play an important role. Cigarette smoking increases the risk of both cardia and non-cardia tumors, whereas high intake of salted or salt-preserved foods has been associated with a higher incidence of non-cardia cancer [17,18]. GC remains more common in males than females, a disparity potentially explained by sex-specific prevalence of risk factors or hormonal influences [19].

The proportion of patients with a resectable disease also varies widely. Population-based European studies show that, among non-metastatic GC cases, approximately 60–70% undergo curative-intent gastrectomy. However, resection rates differ considerably across countries, reflecting variability in stage at diagnosis, availability of high-volume surgical centers, patient characteristics, and evolving treatment paradigms [20,21]. Accordingly, despite advances in treatment, the prognosis remains poor. In most countries, the 5-year survival rate is approximately 20%, in stark contrast to the substantially higher rates reported in Japan (65%) and South Korea (71.5%), where population-based endoscopic screening programs facilitate early detection [22,23].

These data underline the primary potentially prognostic impact of endoscopic strategies by detecting precancerous conditions and EGC.

### 2.2. Precancerous Conditions

#### 2.2.1. Chronic Atrophic Gastritis and Gastric Intestinal Metaplasia

Chronic atrophic gastritis (CAG) and gastric intestinal metaplasia (GIM) are precancerous conditions, as they represent the principal histological substrate in which dysplasia and intestinal-type adenocarcinoma arise. Nevertheless, they independently confer an increased risk of developing GC. According to the Correa cascade, chronic gastric inflammation evolves through atrophic gastritis and intestinal metaplasia, eventually leading to dysplasia and intestinal-type GC [4]. The diagnosis and grading of CAG rely on the evaluation of chronic inflammatory infiltration, characterized by lymphocytes and plasma cells expanding the lamina propria, as well as on the disappearance of native gastric glands. In the gastric body and fundus, these changes correspond to a loss of specialized cells, resulting in reduced gastric secretory function [24]. The degree of glandular atrophy should be carefully assessed. Gastric atrophy is a condition characterized by the loss of native epithelium, which may be replaced by fibrotic tissue (non-metaplastic atrophy) or by a different type of epithelium (intestinal metaplasia or pseudopyloric metaplasia) [25]. If this pathological process occurs in the corpus, the subsequent reduced mass of specialized parietal cells leads to impaired gastric acid and intrinsic factor secretion, eventually resulting in malabsorption of iron and vitamin B12 and consequent anemia. This condition is also linked to an increased risk of GC as a consequence of the changed intragastric microenvironment [26]. Nevertheless, the continuous stimulation of antral gastrin-producing cells with endocrine-like cell hyperplasia may lead to the development of type 1 gastric neuroendocrine tumors [27]. Gastric intestinal metaplasia (GIM) is an advanced stage of atrophy since intestinal-type glands appear after losing gastric glands [4]. GIM can be categorized as ‘complete’ (or type I), ‘incomplete’ (or type II), and a more undifferentiated type (or type III). Complete GIM is characterized by mature absorptive cells, goblet cells, and a brush border, reflecting a more differentiated small-intestinal phenotype. Incomplete GIM resembles colonic epithelium and expresses both intestinal and gastric mucins. Type III GIM, finally, is a more undifferentiated type of GIM. A multicenter study from Spain showed that incomplete GIM carries a higher risk than complete GIM of progressing to GC, thus underlying relevant clinical implications of this classification [28].

Gastric atrophy and GIM severity can be staged by using the Operative Link on Gastritis Assessment (OLGA) and Operative Link on Gastric Intestinal Metaplasia (OLGIM) classifications. These scoring systems were developed to standardize the histological reporting of chronic gastritis and intestinal metaplasia by adding a prognostic, cancer-risk-based staging component to traditional biopsy evaluation, incorporating atrophy and GIM scores (assign stages 0–IV) from both the antrum and corpus to create stages that reflect the extent of mucosal atrophy, which correlates with GC risk. By providing a clear, internationally accepted staging method, OLGA improves communication among clinicians, increases interobserver consistency, and offers a more practical tool for routine biopsy reporting and patient management [29]. Overall, OLGIM is preferred over OLGA when cancer-risk prediction depends on the presence and extent of intestinal metaplasia [30]. However, higher stages in OLGA and/or OLGIM systems are associated with a significantly increased risk of developing high-grade dysplasia (HGD) and GC, thereby validating these scoring systems for GC risk stratification and for the design of endoscopic surveillance programs [31]. Current evidence suggests that virtual chromoendoscopy (VCE) with narrow-band imaging (NBI) provides high diagnostic accuracy for detecting gastric precancerous conditions. An endoscopic classification system, the Endoscopic Grading of Gastric Intestinal Metaplasia (EGGIM), was introduced to improve GC risk stratification through real-time endoscopic assessment of GIM. This classification evaluates five gastric regions: the antrum (lesser and greater curvature), the incisura, and the corpus (lesser and greater curvature), using high-resolution endoscopes with NBI [32]. Each area is assigned a score of 0 (absence of GIM), 1 (focal GIM involving ≤30% of the examined area), or 2 (extensive GIM involving >30%), resulting in a cumulative score ranging from 0 to 10. The EGGIM system has shown good agreement with histological assessment of GIM and has demonstrated clinical usefulness in risk stratification, as patients with scores ≥ 5 can be reliably selected for endoscopic surveillance [33]. Similarly, the Kimura–Takemoto endoscopic classification enables assessment of gastric neoplasia risk using white-light endoscopy by categorizing gastric atrophy into closed types (C0–C3) and open types (O1–O3) and further stratifying it into three grades: mild (C1–C2), moderate (C3–O1), and severe (O2–O3). Patients with severe or open-type endoscopic gastric atrophy at baseline should undergo close surveillance to early detect premalignant lesions and cancer. Based on these findings, EGGIM and Kimura–Takemoto have been proposed as safe, cost-effective, and environmentally sustainable alternatives to biopsy-based surveillance strategies, although appropriate training in their application remains necessary [34]. Given these characteristics, the integration of artificial intelligence (AI) in the assessment of these classifications appears particularly promising [35,36]. AI-assisted scoring could provide objective, reproducible per-patient evaluations, as long as implementation follows rigorous technical and clinical standards. Such an approach may enhance diagnostic accuracy, facilitate broader adoption in routine practice, and significantly reduce the need for gastric biopsies [37].

HP infection represents the principal initiating factor of the Correa cascade and a central target for both primary and secondary prevention of gastric cancer. Accumulating evidence indicates that successful HP eradication can halt the progression of chronic gastritis and may lead to partial regression of gastric atrophy, particularly when treatment is performed in the early stages of mucosal damage. In contrast, the reversibility of gastric intestinal metaplasia appears to be more limited, although eradication therapy may still reduce the risk of further progression along the carcinogenic cascade [38,39]. Importantly, several longitudinal studies and meta-analyses have demonstrated that HP eradication significantly reduces the incidence of gastric cancer, especially when implemented before the development of advanced precancerous lesions [40]. Therefore, according to current international consensus reports [41], HP eradication is strongly recommended in patients with chronic atrophic gastritis and intestinal metaplasia as part of the management of precancerous gastric conditions and strategies for gastric cancer prevention and early detection.

#### 2.2.2. Dysplasia

Dysplasia, also referred to as intraepithelial or non-invasive neoplasia, is defined by the WHO as neoplastic epithelium lacking stromal invasion and characterized by cytologic atypia and architectural disorganization [42]. Typically, dysplasia follows CAG and GIM in the sequential model proposed by Correa; thus, it is more frequent in patients with those precancerous conditions. According to the meta-analysis by Akbari et al. [43], the incidence of new gastric dysplasia was estimated at 6.23 cases per 1000 person-years (95% CI 2.34–11.46) among patients with atrophic gastritis, and at 12.51 cases per 1000 person-years (95% CI 5.45–22.03) among those with GIM. The endoscopic prevalence of gastric dysplasia varies from 0.5% to 3.7% in Western countries and from 9% to 20% in areas with a high incidence of gastric adenocarcinoma. Beyond its prevalence, dysplasia is clinically relevant because it confers a substantial risk of gastric adenocarcinoma (40.4 cases per 1000 person-years). Since dysplastic cells undergo sequential changes from well-differentiated to poorly differentiated, dysplasia is classified as low-grade or high-grade, reflecting the cancer risk of each phenotype [44]. Short-term studies on the natural history of gastric dysplasia showed that patients with HGD had a rate of malignant progression or synchronous malignant lesions of 60–85% over a median interval period of 4–48 months. The risk of progression in individuals with LGD is less clear. There is evidence to show that LGD will regress in 38–75% of patients and persist in 19–50%. In the LGD lesions that persist, the risk of malignant progression ranges from 0% to 23% in the published literature over 10–48 months [45]. Long-term cumulative risk estimates demonstrate that LGD carries a 5–10-year carcinoma risk of roughly 2–6%, whereas HGD is associated with substantially higher cumulative incidence, often exceeding 20–30% over similar intervals [46]. However, the progression risk of gastric dysplasia does not follow a linear pathway but can be influenced by some additional elements. First, HP infection is a critical modifier of risk, as persistent infection increases the likelihood of advancement along the Correa cascade, while successful eradication reduces both dysplasia progression and metachronous neoplasia [39]. Nevertheless, other clinical and endoscopic predictors consistently correlating with progression include older age, male sex, lesion size, depressed morphology, proximal location, and family history of gastrointestinal (GI) cancer. Smoking is a dose-dependent risk factor for GC and combined with alcohol further increases the risk. Consequently, effective preventive measures could significantly reduce the incidence of GC [47]. The histopathological biopsy diagnosis may not be representative of the final histopathological grade on excision. It is not unusual to find a report “indefinite for dysplasia” when histology shows borderline characteristics. In follow-up evaluations, these cases demonstrate upgrade rates of LGD in 7%, HGD in 5%, and invasive carcinoma in 23–29%. Overall, up to 40% of cases show histologic upgrade on subsequent biopsy or endoscopic resection. These data reinforce that even borderline or indeterminate lesions can harbor or evolve into more advanced neoplastic changes [48,49]. Another element to consider is that biopsy-based grading of lesion-associated dysplasia frequently underestimates neoplastic severity. Accordingly, a meta-analysis including lesions with a histologic diagnosis of LGD showed an upgrade to HGD or intramucosal carcinoma in approximately 25% of cases following endoscopic resection, particularly in lesions > 10–20 mm or with depressed or irregular morphology [50]. Lastly, it is necessary to underline that the Correa cascade is not always applicable. Indeed, some evidence highlights a discrepancy between white-light endoscopy and histological biopsy, indicating that dysplasia may be macroscopically ‘non-visible’ and may potentially arise from a not severely atrophic mucosa [51]. Nevertheless, although elevated OLGA/OLGIM stages undoubtedly correlate with a higher likelihood of dysplasia and GC, several reports have documented cases of gastric dysplasia or adenomas arising in otherwise normal or only minimally altered mucosa, including foveolar-type adenomas developing in non-atrophic background mucosa and even high-grade dysplasia occurring in HP-negative, non-atrophic stomachs [52].

### 2.3. Endoscopy in Precancerous Gastric Lesions

Given the previously reported increased GC risk in certain gastric epithelial alterations, several endoscopic follow-up protocols have been proposed. Of these, the most comprehensive are outlined in the MAPS III guidelines [53]. These guidelines consider endoscopic and histologic findings, as well as patient-specific characteristics, to determine the necessity and frequency of endoscopic surveillance (Table 1). Regarding gastric dysplasia, a distinction must be made between lesion-associated dysplasia and dysplasia not associated with an endoscopically recognizable lesion. Dysplasia without an endoscopically visible lesion should undergo a repeat high-quality endoscopy with VCE, staging biopsies, and HP testing; if no lesion is again identified, surveillance is recommended at 6 months for high-grade dysplasia and at 12 months for low-grade or indefinite dysplasia. Conversely, when an endoscopically visible lesion harboring dysplasia is present, endoscopic staging with optical diagnosis (with or without targeted biopsies) and endoscopic-based treatment is recommended, mainly performed through endoscopic submucosal dissection (ESD), being the treatment of choice for most superficial gastric lesions (Table 2).

### 2.4. Interval Gastric Cancer

Despite the endoscopic follow-up protocols in high-risk groups, current data show a suboptimal efficacy of EGD to prevent GC. Indeed, interval gastric cancer, meaning GC diagnosed between a “negative for cancer” endoscopy and the next scheduled EGD, represents a non-negligible phenomenon that deserves attention. A recent study conducted in South Korea found that among patients who developed GC within 6–36 months after an index EGD considered negative for malignancy, about 8.1% of the cases were cancers at advanced stages [6]. Another investigation, using a different cohort and defining interval cancers as those detected 2–3 years after a prior negative EGD, reported that 18.4% of GCs diagnosed in that period met criteria for so-called “interval gastric cancers” [54]. In a United States-based series analyzing 751 patients with upper-GI malignancies (esophageal or gastric), 52 (6.9%) patients were found to have cancers emerging 6–36 months after an EGD that had been negative for cancer [55]. Various risk factors appear to be associated with an increased likelihood of interval gastric cancer after a negative EGD. Among these, a shorter observation time during endoscopy has been identified as significant [6,54]. Other contributing factors include the presence of precancerous gastric changes (such as gastric atrophy or intestinal metaplasia), initial lesions that are flat or ulcerated and thus easily missed, and localization of lesions in challenging anatomical zones, specifically the low-to-mid body of the stomach, where visibility may be reduced and blind spots are more common [56]. These findings highlight a crucial limitation of endoscopic screening: whereas a “negative for cancer” endoscopy cannot entirely exclude the possibility of a subsequent diagnosis, either because the cancer was missed at the time or because it developed de novo in the intervening period cannot entirely exclude the possibility of a subsequent diagnosis.

In this context, it is important to distinguish between missed cancers and so-called “true” interval gastric cancers, which arise de novo between scheduled examinations. True interval cancers are frequently associated with undifferentiated histology, particularly diffuse-type gastric cancer and poorly cohesive or signet-ring cell carcinoma [42]. These tumors are characterized by an infiltrative growth pattern with minimal mucosal architectural distortion and early submucosal spread. Due to their growth characteristics, these lesions may exhibit subtle or non-specific endoscopic findings, sometimes mimicking inflammatory changes such as gastritis, and may progress more rapidly than intestinal-type gastric cancer [57]. This biological behavior may explain their occurrence as interval cancers despite apparently adequate surveillance. Nevertheless, even after endoscopic treatment of HGD or EGC, patients remain at increased risk of metachronous EGC, (which may arise as early as 15 months after the initial lesion) [56], since they are associated with a substantial risk of synchronous neoplasia. Finally, these points underscore the importance of high-quality endoscopic technique and possibly closer surveillance in patients with known risk factors (atrophy, metaplasia) or other predisposing conditions.

Although it lies somewhat beyond the primary scope of the present review, special clinical settings also deserve consideration, as early-onset gastric cancer and hereditary gastric cancer syndromes may require risk-based screening and surveillance strategies [58], whereas signet-ring cell carcinoma and poorly differentiated gastric cancer may be highly occult at endoscopy, thus requiring a more accurate endoscopic inspection [59]. Indeed, a small subset of gastric cancers, such as diffuse-type, poorly cohesive, or signet-ring cell-type, may remain underdiagnosed even during high-quality endoscopy, owing to infiltrative growth with non-specific endoscopic features [60].

## 3. Quality Parameters in Upper-GI Endoscopy

### 3.1. The Pursuit of Quality

Given the evidence mentioned above, particularly in the esophagus and stomach, performing an EGD to provide a high-quality diagnostic procedure is necessary. The first step in optimizing quality is to “speak the same language”, meaning to standardize endoscopy reports as an integral component of quality enhancement in EGD. This involves knowing “what” and “how” to describe each endoscopic finding in a structured and reproducible way [61]. The second step, which will be deeply discussed below, is the achievement of specific quality markers that should be embedded into routine endoscopic practice.

Unfortunately, the adherence to quality indicators in EGD is generally lower than in colonoscopy, due to a non-uniform application across different endoscopy centers [62]. In an Italian national survey, Zagari et al. [63] showed that almost all endoscopists record key colonoscopy parameters (Boston Bowel Preparation Scale, cecal intubation rate ≥90%), whereas for EGD only 18.2% record the procedure duration and only half provide photographic documentation for most of their procedures. Consequently, the rate of missed upper-GI cancers still remains high [64]. For this reason, several studies [65] defined standard approaches that can guide clinical practice from pre-procedural assessment to discharge time. Appropriate indications, tailored time slots, systematic use of premedication, and HD endoscopy with advanced imaging to optimize mucosal visualization and pictures of key landmarks are some of the required practices that should be part of EGD practice. Altogether, these developments are shifting EGD toward a more measurable, auditable, and cancer-focused quality paradigm [66].

### 3.2. Quality Performance Measures

Recently, ESGE updated the performance measures in this field [67], redefining the statements aimed at the early detection of neoplastic lesions. The update introduces validated mucosal visibility scores as a required reporting item and reinforces the minimum examination time of ≥7 min from intubation to scope withdrawal. Photodocumentation is better standardized, with a defined set of 10 landmarks plus all the findings of interest. Moreover, the concept of quality is extended “outside” the scope by recommending time slots ≥ 20 min for diagnostic EGD to allow careful inspection and reporting. Compared with the 2016 document [68], major novelties are the inclusion of surveillance adherence for precancerous conditions as key performance measures [53] and, for the first time, the integration of patient-reported experience instruments as a formal quality domain, underscoring that high-quality EGD must be both diagnostically effective and patient-centered.

### 3.3. Observation Time and Photodocumentation

Observation time has always been recognized as an important quality indicator in EGD. More dedicated time means more careful mucosal inspection, representing one of the key parameters of quality in EGD. This element is driven by the significant increase in the detection rate of pre-neoplastic and neoplastic lesions. Indeed, several studies [69,70] have demonstrated a direct proportional relationship between observation time and the detection of focal gastric lesions, with a gap between slower and faster endoscopists that can reach about 5% (*p* < 0.0001) in some series [71]. Moreover, a longer observation time is strictly linked to a higher number of pictures, which are taking on an increasingly important role according to the latest guidelines, as explained above. The importance of photodocumentation also implies greater emphasis on optical assessment. Indeed, as proposed by several authors, a large collection of gastric images could minimize the missing rate [72]. In particular, in a retrospective study enrolling more than 42,000 EGD [73], Kashiwagi K et al. found a significant negative relationship between the gastric neoplasm detection rate and the number of gastric images < 35 (OR 0.305, 95%CI 0.189–0.492; *p* < 0.001), suggesting that the lower the number of images, the lower the detection rate. Moreover, in their study, Kashiwagi K et al. suggest that a total of 50 images is necessary for detecting upper-GI cancers.

### 3.4. Gastric Cleansing and Mucosal Visibility Scores

Another emerging factor is gastric cleansing. Although most authors [74] agree on the effectiveness of simethicone (SIM) plus N-acetylcysteine (NAC) [75,76] to provide the best cleaning of the gastric mucosa, fewer data are available on the timing of SIM administration. Only one multicenter single-blind RCT [77] aimed to evaluate any differences between timing of administration, dividing patients into three groups (early group: patients who received SIM 20–30 min before the procedure; late group: patients who received SIM 0–10 min before the procedure; and split-dose group including patients who received half-dose at both time points). Percentages of adequate total mucosal visibility were 55%, 42%, and 61% in the early, late, and split-dose groups, respectively (*p* < 0.01). Therefore, it can be concluded that the optimum to achieve the best cleansing is at least one early SIM administration. Adequate gastric cleansing, as occurs for colonoscopy, could potentially increase diagnostic yield. Reasonably, the detection rate of an EGD performed under inadequate gastric cleansing cannot match that of an EGD under optimal conditions. In addition, several validated mucosal visibility scores have been proposed; the most widely used and guideline-recognized are GRACE (Gastroscopy RAte of Cleanliness Evaluation) [78], which assigns a score to all EGD-assessable segments, including the esophagus, stomach, and duodenum. The PEACE (Polprep: Effective Assessment of Cleanliness in EGD) score [79,80] and the Barcelona scale [81] are also validated tools, which typically assess the presence of mucus, bubbles, and residual fluid using ordinal scales to quantify visibility. These scores, together with others that warrant further external validation [82], may represent a further step toward improving the standardization of endoscopic reports.

## 4. Beyond Endoscopy: Chromoendoscopy and Magnification

### 4.1. Endoscopic Staging

The image, namely the adequate endoscopic observation, is increasingly becoming the emerging cornerstone in EGD quality. Similarly, MAPS III guidelines [53] have enhanced the key role of endoscopy by adopting an endoscopy-based staging strategy for the management of these premalignant changes in the stomach. In particular, the EGGIM [33] and Kimura–Takemoto classifications [83] have assumed, in the latest update, a pivotal role for decision making regarding endoscopic follow-up of precancerous lesions. This direction enhances the utility of accurate assessment of the gastric mucosa, reserving bioptic sampling for dysplastic lesions.

### 4.2. Virtual Chromoendoscopy

**Figure 2:**
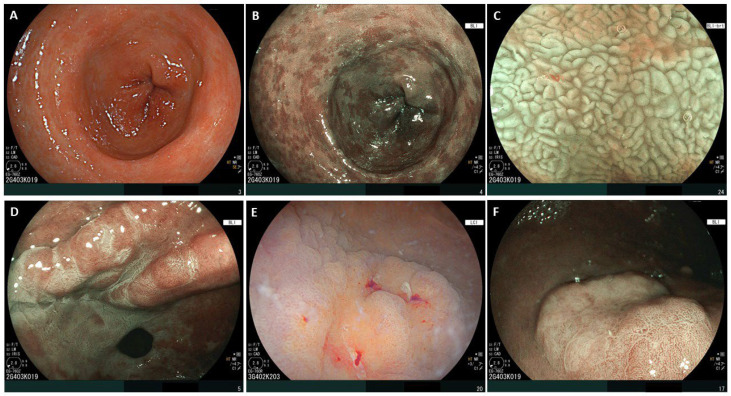
Panel (**A**): White-light imaging (WLI)—Antrum: The antral mucosa shows only subtle dyschromic areas, making an accurate, definitive diagnosis challenging with WLI alone. Panel (**B**): Blue-light imaging (BLI) mode—Antrum: Panoramic assessment with virtual chromoendoscopy (VCE) reveals diffuse whitish plaques. Panel (**C**): Magnified blue-light imaging (M-BLI)—Antrum: Antral mucosa with extensive intestinal metaplasia, including some specific characteristics: villiform appearance and white opaque substance (WOS) with regular assessment. Panel (**D**): Image obtained with combination of VCE (BLI) and acetic acid 1%—Lesser curvature of the antrum, anterior wall: Intestinal metaplasia mixed in with normal antral mucosa. Intestinal metaplasia shows a transient aceto-whitening reaction. Panel (**E**): Linked color imaging (LCI) evaluation of dysplastic lesion—Greater curvature of the antrum, proximal side: An orange coloration is a typical feature of dysplastic lesions or early gastric cancer. In this figure, focal areas of spontaneous bleeding are also observed, which is another characteristic sign of a neoplastic lesion. Panel (**F**): BLI evaluation of displastic lesion—Greater curvature of the antrum: Pseudodepressed, with irregular vessels, avascular areas, macrovessels, mildly elevated border and demarcation line, surrounded by antral and metaplastic mucosa. The figure originate from examinations performed at the authors’ institution and merely illustrates established knowledge, mostly accessible in the MAPS III document from Dinis Ribeiro et al. [53].

Recently, new technologies have been offering added value to optical diagnosis. In this setting, VCE (through the use of NBI, blue light imaging (BLI), linked color imaging (LCI), or i-scan software), and magnification are the most common available tools. With these methods, visualization of epithelial and vascular patterns is improved, facilitating metaplastic and dysplastic lesion assessment. Indeed, it is now widely recognized that assessment in white light alone may not be sufficient for the detection of neoplastic or non-neoplastic lesions, as it has been largely surpassed by novel image processing technology (Figure 2) [84]. In 2020, a meta-analysis by Rodriguez-Carrasco et al. [85] demonstrated the high specificity of VCE using NBI in GIM detection, ranging from 0.91 (95%CI: 0.88–0.94) to 0.95 (95%CI: 0.94–0.96), respectively, in per-patient or per-lesion based analysis. On the other hand, performance metrics in dysplastic lesions were sensitivity 0.87 (95%CI: 0.84–0.89) and specificity 0.97 (95%CI: 0.97–0.98). Moreover, they concluded that studies using magnified endoscopy had a higher diagnostic performance, suggesting chromoendoscopy-plus-magnification combined use in clinical practice. Among the different VCE software, the use of BLI has also been shown to be non-inferior to the diagnostic accuracy provided by NBI technology [86]. More specifically, the diagnosis of GIM is enabled by the assessment of two key characteristics, a light-blue crest (LBC) and white opaque substance (WOS) (Figure 2C). LBCs are blue-whitish patchy areas observable in chromoendoscopy–magnification endoscopy on the gastric epithelial surface. The presence of this feature allows GIM diagnosis with a sensitivity of 0.90 (95%CI: 0.86–0.92) and a specificity of 0.90 (95%CI: 0.86–0.93) [87]. WOS is a phenomenon that occurs when microscopic lipid droplets are stocked within the GIM epithelium, with a sensitivity and specificity for GIM of 0.50 (95%CI: 0.40–0.50) and 1.00 (95%CI: 0.85–1.00), respectively [88]. LBC and WOS are both useful markers for endoscopic diagnosis of GIM, as is the improved effect provided by their combined evaluation. Nevertheless, starting from these two features, it is possible to build endoscopic classifications such as the vessel-plus-surface (VS) classification system, an established diagnostic system developed by Yao et al. [89], which allows differentiation between neoplastic and non-neoplastic lesions. The VS classification system is based on microvascular assessment, including the description of the subepithelial capillary network, collecting venules and microvessels (MVs), and on the microsurface (MS) pattern, including marginal crypt epithelium, crypt opening, the intervening part between crypts, LBCs, and WOS. Simplifying this classification, the presence of an irregular microvasculature and/or an irregular microsurface pattern with a demarcation line supports the diagnosis of a neoplastic lesion. The demarcation line itself may also show peculiar features, e.g., a multiple convex demarcation line, which is protruded toward the inside, has been shown to have a high specificity (91%) and positive predictive value (97%) for non-neoplastic lesions [90]. LCI can also provide added value to endoscopic visualization. In LCI mode, a deep purple color is associated with GIM plaques, whereas dysplastic lesions typically show an orange-red, orange, or orange-white surface (Figure 2E) [91].

### 4.3. Magnifying Endoscopy

In clinical practice, a highly reproducible diagnostic algorithm is the magnifying endoscopy simple diagnostic algorithm for gastric cancer (MESDA-G) [92], which involves the assessment of any suspicious lesions by describing, using magnifying evaluation, first the presence and characteristics of a demarcation line and subsequently MV and MS patterns separately, as regular, irregular, or absent. This provides a high accuracy (95%), a high positive predictive value (79%), and a high negative predictive value (99%) for neoplastic lesions [93]. However, this approach has some limitations. Most false-negative cases are caused by clinicopathological features of the cancer, i.e., well-differentiated adenocarcinoma with low-grade atypia, as happens in fundic-gland type adenocarcinoma, or lesions that are partially or completely covered by non-neoplastic mucosa [94]. This underscores the incomplete understanding of GC pathophysiology and helps explain why some authors advocate for a more holistic approach to patient care [95].

### 4.4. Acetic Acid

Another tool available to improve diagnostic accuracy is acetic acid. Indeed, it allows the identification of GIM and dysplastic mucosa through differences in aceto-whitening reaction [96,97]. The mechanism consists of an enhancing effect on columnar epithelium with an intestinal phenotype, thanks to its ability to reversibly denature cellular proteins of the columnar epithelium, particularly cytokeratins, thus increasing mucosal opacity and generating the aceto-whitening phenomenon [98]. GIM and gastric dysplasia show different whitening patterns and disappearance times compared with normal mucosa [99]. Acetic acid also allows the distinction between neoplastic and non-neoplastic (or metaplastic) epithelium, thanks to the different duration and intensity of the aceto-whitening reaction, as well as a better delineation of lesion margins (Figure 2D) [100]. The therapeutic implication is reflected in an improved assessment of the lateral margins of neoplastic lesions, also in view of ESD [101].

### 4.5. Bioptic Sampling

The recognizability of epithelial and vascular patterns through magnification and chromoendoscopy enables reliable optical diagnosis, with high sensitivity and specificity for dysplasia, reducing the need for random biopsies [102]. Given the high diagnostic accuracy of those optical diagnosis techniques, it is the authors’ opinion that the integration of advanced techniques will progressively reduce reliance on random sampling. As yet, guidelines do not endorse a bioptic-free approach; however, targeted biopsies guided by advanced imaging reduce sampling error and the total number of biopsies needed. They not only increase diagnostic accuracy but also reduce the number of samples, procedural cost, and environmental footprint [103]. In addition, many authors believe that the major shift will consist in overcoming the traditional practice of random biopsies, limited by sampling error and poor adherence, in favor of a visual-based approach and chromoendoscopy-target sampling [104].

In conclusion, as a systematic and sustainable approach for a high-quality clinical practice, the authors believe that every EGD should include a VCE assessment, possibly combined with magnified endoscopy.

### 4.6. The Role of Artificial Intelligence

AI is emerging as a relevant adjunct in upper-GI diagnosis, with potential applications that extend well beyond simple neoplastic lesion detection. Indeed, AI-based quality assurance systems can support real-time recognition of gastric landmarks, monitor blind spots, and improve the completeness of EGD. In a multicenter randomized trial [105], ENDOANGEL significantly reduced the number of blind spots and increased inspection time, with a significant reduction in gastric neoplasm miss rate, as demonstrated in a tandem RCT [106]. More recently, automated photodocumentation systems have also shown improvement in both completeness and image quality during examinations [107]. Regarding optical diagnosis, AI has shown promising potential both in the setting of premalignant gastric lesions, as reported by a recent meta-analysis [108], which found a pooled sensitivity and specificity of 94% and 93%, respectively, and in the detection of neoplastic lesions. Indeed, AI-based tools have also been shown to assist in the detection of subtle early neoplastic lesions even under white-light endoscopy, potentially narrowing the performance gap between expert and non-expert endoscopists. Lastly, a prospective study based on probe-based confocal laser endomicroscopy [109] demonstrated expert-level, whole-chain AI diagnosis across the Correa cascade, with high accuracy for inflammation, atrophy, GIM, and neoplastic lesions, while also suggesting a possible reduction in unnecessary biopsies. Despite these encouraging results, most currently available data derive from expert centers, while the most recent guidelines still consider the evidence insufficient to recommend routine AI adoption.

## 5. Toolkit for Good Practice

**Figure 3:**
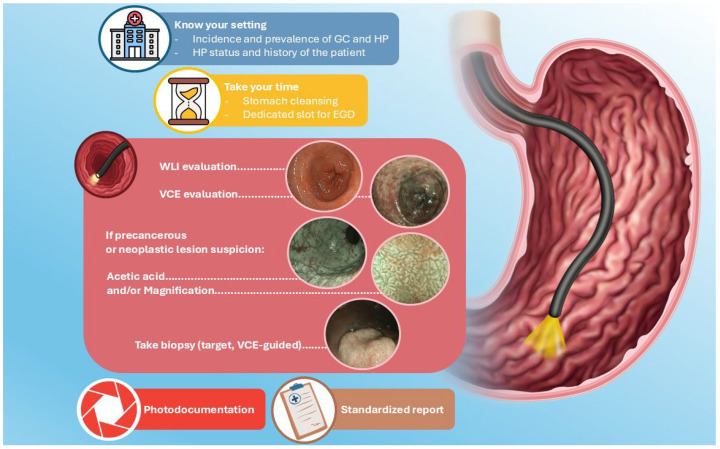
Toolkit for endoscopists. GC: Gastric cancer; HP: Helicobacter pylori; EGD: esophagogastroduodenoscopy; WLI: white-light imaging; VCE: virtual chromoendoscopy.

The scientific evidence described above allows the definition of good clinical practice for the endoscopist, identifying the tools that enable the most effective diagnostic approach in upper-GI endoscopy. Figure 3 summarizes the key steps discussed in this review, beginning with a thorough understanding of the clinical context in which the procedure is performed and the adequate time allocation for EGD, and extending to careful assessment using advanced endoscopic techniques (VCE, magnification, acetic acid). The authors’ intent is to provide a practical toolkit that, when applied step by step, may enhance the quality of EGD and reduce the miss rate of precancerous lesions.

## 6. Conclusions

GC continues to represent a significant global health burden, despite a steady decline in incidence rates. This downward trend is largely attributable to improvements in living conditions, decreasing prevalence of HP infection, and modifications in dietary habits and environmental exposures. Nevertheless, the absolute number of GC cases is projected to rise in the coming years, mainly driven by population aging and demographic growth in high-risk regions. This apparent epidemiological contradiction highlights the persistent need for effective prevention measures, as well as optimized strategies for early detection and endoscopic surveillance. Countries such as Japan and South Korea, where population-based endoscopic screening programs have been widely implemented, report substantially higher proportions of EGC and markedly improved survival rates compared with Western countries. Early detection through endoscopic screening represents the most effective strategy to reduce cancer-related mortality and improve long-term outcomes. However, the effectiveness of endoscopic screening depends not only on its implementation but, critically, on the quality of the examination itself. High-quality EGD must be regarded as a complex, operator-dependent procedure requiring sufficient inspection time, systematic mucosal evaluation, and adherence to standardized quality indicators. Endoscopist expertise plays a central role in lesion recognition, optical diagnosis, and appropriate management decisions, directly influencing diagnostic accuracy and the risk of missed neoplastic lesions. At the same time, continuous technological advancements have significantly enhanced the diagnostic capability of endoscopy, facilitating earlier detection of precancerous and neoplastic lesions. The integration of these technologies into routine clinical practice, combined with structured training and quality-oriented protocols, represents a key step toward optimizing the effectiveness of endoscopic screening. Future efforts should focus on expanding access to high-quality endoscopy, standardizing performance measures, and promoting continuous training, in order to maximize early detection and improve outcomes for patients with gastric neoplasia.

## Figures and Tables

**Table 1:** Endoscopic surveillance of gastric precancerous lesions according to the MAPS III guidelines [53].

Endoscopic Surveillance of Gastric Precancerous Lesions			
OLGA/OLGIM 0–II **AND** No iGIM AND C0–2 **AND** EGGIM 0–4 **AND** No first-degree relative with gastric cancer **AND** No HP infection	Extensive GIM **OR** OLGA/OLGIM III–IV or iGIM **OR** C3+/EGGIM 5+ **AND** First-degree relative with gastric cancer	Extensive GIM **OR** OLGA/OLGIM III–IV	LGD
		**OR** iGIM **OR** C3+/EGGIM 5+	HGD
		**OR**	
		OLGIM I–II + First-degree relative with gastric cancer	Indefinite for dysplasia
		**OR**	
		HP persistence	
**No surveillance (return to screening if applicable)**	**Surveillance at 1–2 years**	**Surveillance at 3 years**	**Reassess in 6–12 months (HGD/LGD)**

LGD: Low-grade dysplasia; HGD: high-grade dysplasia; GIM: gastric intestinal metaplasia; OLGA: Operative Link on Gastritis Assessment; OLGIM: Operative Link on Gastric Intestinal Metaplasia; iGIM: incomplete-GIM; EGGIM: Endoscopic Grading of Gastric Intestinal Metaplasia; C: Kimura–Takemoto classification; HP: Helicobacter Pylori.

**Table 2:** Endoscopic surveillance and management of gastric dysplasia associated or not with an endoscopically visible lesion.

Endoscopic Management	Recommended Follow-Up Interval	Clinical Scenario
Repeat high-quality endoscopy + VCE + staging biopsies	6 months (HGD)/12 months (LGD or indefinite)	Dysplasia without an endoscopically visible lesion
Surveillance with VCE and biopsies only from suspicious areas	3–6 months, then annually	Post-resection (EMR/ESD) of a visible dysplastic lesion (R0 margins)
Repeat endoscopic resection or consider surgical evaluation	Early multidisciplinary evaluation	Post-resection (EMR/ESD) of a visible dysplastic lesion with non-R0 margins or residual dysplasia

LGD: Low-grade dysplasia; HGD: high-grade dysplasia; EMR: endoscopic mucosal resection; ESD: endoscopic submucosal dissection; VCE: virtual chromoendoscopy.

## Data Availability

No new data were created or analysed in this study. Figure 2 was generated from Management of epithelial precancerous conditions and early neoplasia of the stomach (MAPS III): European Society of Gastrointestinal Endoscopy (ESGE), European Helicobacter and Microbiota Study Group (EHMSG) and European Society of Pathology (ESP) Guideline update 2025, as reported by author Dinis Ribeiro et al. [53]. Data sharing is not applicable to this article.

## Abbreviations

The following abbreviations are used in this manuscript:

GIM	Gastric Intestinal Metaplasia
HP	Helicobacter Pylori
EGC	Early Gastric Cancer
CAG	Chronic Atrophic Gastritis
ESGE	European Society of Gastrointestinal Endoscopy
LBC	Light-Blue Crests
WOS	White Opaque Substance
OLGA	Operative Link on Gastritis Assessment
OLGIM	Operative Link on Gastric Intestinal Metaplasia Assessment
EGGIM	Endoscopic Grading of Gastric Intestinal Metaplasia
EMR	Endoscopic Mucosal Resection
ESD	Endoscopic Submucosal Dissection
SIM	Simethicone
NAC	N-acetylcysteine
RCT	Randomized Controlled Trial
GRACE	Gastroscopy RAte of Cleanliness Evaluation
PEACE	Polprep: Effective Assessment of Cleanliness in EGD
VCE	Virtual Electronic Chromoendoscopy
NBI	Narrow-Band Imaging
BLI	Blue-Light Imaging
LCI	Linked Color Imaging
VS	Vessel plus Surface
MV	Microvessels
MS	Microsurface
MESDA-G	Magnifying Endoscopy Simple Diagnostic Algorithm for Early Gastric Cancer
EGD	Esophagogastroduodenoscopy
GC	Gastric Cancer
